# Western corn rootworm pyrethroid resistance confirmed by aerial application simulations of commercial insecticides

**DOI:** 10.1038/s41598-019-43202-w

**Published:** 2019-04-30

**Authors:** Dariane Souza, Bruno C. Vieira, Bradley K. Fritz, Wesley C. Hoffmann, Julie A. Peterson, Greg R. Kruger, Lance J. Meinke

**Affiliations:** 1 University of Nebraska-Lincoln, Department of Entomology, Lincoln, 68583 USA; 2 University of Nebraska-Lincoln, Department of Agronomy and Horticulture, North Platte, 69101 USA; 3 USDA-ARS, Aerial Application Technology Research Unit, College Station, 77845 USA; 4 University of Nebraska-Lincoln, Department of Entomology, North Platte, 69101 USA

**Keywords:** Entomology, Biological techniques

## Abstract

The western corn rootworm (*Diabrotica virgifera virgifera* LeConte) (WCR) is a major insect pest of corn (*Zea mays* L.) in the United States (US) and is highly adaptable to multiple management tactics. A low level of WCR field-evolved resistance to pyrethroid insecticides has been confirmed in the US western Corn Belt by laboratory dose-response bioassays. Further investigation has identified detoxification enzymes as a potential part of the WCR resistance mechanism, which could affect the performance of insecticides that are structurally related to pyrethroids, such as organophosphates. Thus, the responses of pyrethroid-resistant and -susceptible WCR populations to the commonly used pyrethroid bifenthrin and organophosphate dimethoate were compared in active ingredient bioassays. Results revealed a relatively low level of WCR resistance to both active ingredients. Therefore, a simulated aerial application bioassay technique was developed to evaluate how the estimated resistance levels would affect performance of registered rates of formulated products. The simulated aerial application technique confirmed pyrethroid resistance to formulated rates of bifenthrin whereas formulated dimethoate provided optimal control. Results suggest that the relationship between levels of resistance observed in dose-response bioassays and actual efficacy of formulated product needs to be further explored to understand the practical implications of resistance.

## Introduction

The western corn rootworm (WCR) *Diabrotica virgifera virgifera* LeConte (Coleoptera: Chrysomelidae) is a major pest of corn *Zea mays* L. in the United States (US) and has adapted over time to many management tactics^1,2^. Broadcast application of organochlorine soil insecticides starting in the late 1940’s became rapidly ineffective with control issues apparent by late 1950’s^3^. Aerial applications of carbamates and organophosphates selected adult WCR for significant levels of resistance in the 1990’s^4–6^. Resistance to transgenic corn producing rootworm-specific Cry toxins from the soil bacterium *Bacillus thuringiensis* (*Bt*) was initially reported for Cry3Bb1 in 2009, mCry3a in 2011, and Cry34/35Ab1 in 2016^7–10^. Furthermore, the cultural practice of corn rotation with a nonhost crop was circumvented in areas of the US eastern Corn Belt by WCR oviposition in nonhost crops indicating the evolution of behavioral resistance^2,11,12^. These events collectively have made WCR management exceedingly difficult.

Although crop rotation is the most recommended tactic to manage the WCR, continuous corn (consecutive planting of corn for ≥ two years) is a common agronomic practice in the US western Corn Belt because under irrigation, continuous corn production is often the most profitable, and corn demand is high for confined livestock and ethanol production^9,12^. However, this intensive system of continuous corn production facilitates build-up of WCR densities over time making WCR management an annual challenge^12–14^. Consequently, aerial applications of pyrethroid and organophosphate insecticides are often used to reduce WCR densities and complement other management tactics^13–15^. In this system, other corn pests like western bean cutworm *Striacosta albicosta* (Smith) and two-spotted spider-mite *Tetranychus urticae* Koch are also managed with the same insecticide classes used to manage rootworms, so WCR adults can be exposed to aerial applications as nontarget insects as well^16–18^. These practices have placed annual selection pressure on WCR populations which has led to field-evolved resistance to pyrethroids in southwestern areas of Nebraska and Kansas^13^.

Laboratory bioassays conducted with WCR populations collected across the US Corn Belt have shown that populations from west of the Missouri River were consistently more tolerant to the active ingredient bifenthrin than eastern populations^13^. Furthermore, cross-resistance and synergism studies performed with field collected bifenthrin-resistant populations suggested that multiple mechanisms of resistance could be involved such as target-site insensitivity and higher activity of detoxification enzymes^14^. Although the levels of bifenthrin resistance found were relatively low, resistance levels were highest in the adult stage^13,14^. Despite having laboratory bioassay data documenting resistance, the efficacy of formulated bifenthrin commonly used in aerial applications to control WCR adults had not been formally evaluated.

Field efficacy of foliar insecticides depends on a combination of several factors such as target susceptibility, exposure, and application technique efficiency. For example, aerial application of insecticides can provide uneven coverage in the corn canopy resulting in insect sublethal exposure and reduced control^19^. Some parameters such as spray carrier volumes, droplet size distribution, crop canopy, and environmental conditions often influence the coverage and uniformity of insecticide deposition which can confound field trial results^20–24^. Carrying out consistent field trials to evaluate the efficacy of insecticide aerial applications on adult rootworms is even more challenging considering the potential interaction of population density and pest movement that may occur in the field^12,25–27^. Thus, methods that minimize factors influencing aerial application performance are necessary to evaluate the impact of WCR pyrethroid resistance on the efficacy of recommended foliar insecticides.

Pyrethroid and organophosphate classes contain many of the insecticides recommended for WCR control. However, enhanced metabolism can confer cross-resistance to structurally related insecticides, which has been observed between pyrethroids and organophosphates for different insect species^28–33^. Thus, the likely involvement of detoxification enzymes in the mechanism of WCR bifenthrin-resistance could impact organophosphate efficacy, which needs further investigation^14^. The past use of organophosphates (e.g. dimethoate) may also contribute to selection of WCR adults with increased detoxification enzyme activity. Resistance to both insecticide classes would greatly reduce alternative insecticide options and impact the integration of these compounds with other management tactics. Indoxacarb represents an insecticide class i.e., the oxadiazines, with unique mode of action that was recently registered by the US Environmental Protection Agency (EPA) for use to control WCR adults in field corn^34,35^. This product was reported to reduce adult WCR populations in the field and may provide a tool to help manage WCR pyrethroid resistance^36^. However, the susceptibility of pyrethroid-resistant WCR to the indoxacarb active ingredient needs to be evaluated.

Therefore, as part of a larger project to characterize WCR pyrethroid resistance^13,14^ and to optimize WCR resistance management and integrated pest management (IPM) programs, the objectives of this study were: (1) to develop a method of simulated aerial application that uniformly reproduces insecticide field deposition; (2) use the simulated aerial application method to assess the performance of bifenthrin and dimethoate commercial formulations against pyrethroid-resistant WCR populations; and (3) to conduct dose-response lab bioassays to estimate and compare the susceptibility of pyrethroid-resistant WCR populations to bifenthrin, dimethoate and indoxacarb active ingredients.

## Methods

### Western corn rootworm populations

Six WCR adult populations were tested during this study. Two non-diapausing populations purchased in 2016 and 2017 from Crop Characteristics, Inc., Farmington, MN (S-Lab1) and French Agricultural Research, Lamberton, MN (S-Lab2), and one field population collected from Saunders County, NE (S-Field) in 2016 were used as pyrethroid-susceptible controls given their high susceptibility to bifenthrin measured in prior bioassays^13^. The Saunders Co. field was located at the University of Nebraska Eastern Nebraska Research and Extension Center, which is surrounded by a large area of continuous corn that had not received insecticide aerial applications for over ten years. Bifenthrin had only been used in that area for soil applications in a few small-plot trials. In 2016, two field populations (R-Field1 and R-Field2) previously confirmed to have field-evolved resistance to bifenthrin^13^ were collected from commercial fields 18 Km apart in Keith County, NE. Each field was in continuous corn production plus annual soil- and aerial bifenthrin applications had been made for at least five consecutive years prior to this study. A third bifenthrin-resistant population tested in 2017 bioassays (R-Lab) was collected from Perkins County, NE in 2014 and then reared in a non-diapause background for nine generations under adult selection with a pre-established bifenthrin diagnostic concentration (LC_99_)^13^. Populations were maintained in the Department of Entomology, University of Nebraska-Lincoln, Lincoln, NE using standard lab rearing procedures and temperature profiles to facilitate egg diapause development and termination^9^.

### Aerial application simulation

#### Cornfield spray deposition

A series of aerial spray applications were performed in a late season cornfield (R5) located just south of Snook, TX (30°28′37.1″N, 96°27′58.7″W) in order to document deposition characteristics that would be expected under typical aerial application conditions. Applications were made using an airplane (AirTractor 402B, Olney, TX) equipped with 40° 10-orifice flat fan nozzles at spray application rates of 18.7 and 46.8 L/ha^37^. The spray solution consisted of water and a 90% non-ionic surfactant (R-11, Wilbur-Ellis Company LLC, Tukwila, WA) at 0.25% v/v. At the 18.7 L/ha application rate, a total of 36 nozzles were used, where nozzles were deflected 15 degrees downward and operated at 317 kPa at an airspeed of 225 Km/h. At the 46.8 L/ha rate, a total of 68 nozzles were used with nozzles deflected at 30 degrees and operated at 413 kPa and at an airspeed of 209 Km/h.

During the applications wind speeds were steady at 4 m/s with a temperature of 30 °C and relative humidity of 75%. Water sensitive papers (WSP) (Syngenta Crop Protection AG, Basel, Switzerland) were placed in the field canopy at three locations denoted as Top, Middle, and Bottom. The Top position was located near the uppermost fully extended leaf with the Bottom near the lowermost extended leaf and the Middle located midway between Top and Bottom positions. WSPs were attached to plants spaced 2 m apart covering a full 20 m application swath using binder clips such that the surface was horizontal to the ground. Applications were performed in two sampling lines (blocks), each with ten WSP (replicates) for each canopy and application rate treatment combination.

After each spray pass replication, WSPs were collected and stored in labeled film negative sleeves. WSPs were digitally scanned at 600 dpi (EPSON Perfection V600) and analyzed for droplet size characteristics using Dropletscan (WRK of Arkansas, LLC., Lonoke, AR), an image processing software package designed for WSP analysis^38,39^. Spray deposition rate (L/ha) and droplet size diameters at which 10, 50, and 90% of the total spray volume is contained in droplets of the specified size or less (DV_0.1_, DV_0.5_, and DV_0.9_, respectively) were measured and reported^40^.

#### Wind tunnel and spray-chamber calibration

In 2016, a low-speed wind tunnel at the Pesticide Application Technology Laboratory (PAT-Lab, University of Nebraska–Lincoln, West Central Research and Extension Center, North Platte, NE) was used to identify ground application nozzles and operating pressures that could effectively simulate the spray deposition collected from the cornfield aerial application. This system has been used for droplet sizing of different nozzle designs, tank solutions and pesticide application scenarios^24,41,42^. Droplet size distribution data collected at Middle corn canopy position were targeted since this would include the major feeding and activity zone for WCR adults in the field^43^. Tap water solutions of Brigade 2EC (bifenthrin 25.1%, FMC Corporation, Philadelphia, PA) were prepared using the highest label rate recommended for rootworm control (112.1 g ai/ha) at the lowest and highest recommended carrier volume rates (18.7 and 46.8 L/ha respectively). The application of the insecticide solutions was then performed in the wind tunnel with a TT110015 ground spray nozzle (TeeJet Technologies, Spraying Systems Co., Wheaton, IL) at different spray pressures until targeted droplet size parameters were achieved. Droplet size distribution data were evaluated by a Sympatec Helos/Vario KR laser diffraction system (Sympatec Inc., Clausthal, Germany) placed at 0.3 m from the nozzle tip to ensure full spray atomization prior to measurement. The system was equipped with an R7 lens that detects droplets in a range from 9 to 3700 μm. Nozzles were attached to an actuator and traversed vertically at a constant speed (0.2 m/s) to ensure that the entire spray plume crossed the laser diffraction system. Volumetric droplet size distribution parameters DV_0.1_, DV_0.5_, and DV_0.9_ were reported. The wind tunnel insecticide applications and respective droplet size distribution measurements were carried out with three replications under controlled conditions of 20 ± 1 °C and 60-70% relative air humidity. Once the best spray pressure for the TT110015 ground spray nozzle was found, the timed output volume per nozzle was measured three times with a graduated glass cylinder (Fisherbrand™, Thermo Fisher Scientific Inc., Waltham, MA, Cat. No. S63461). A customized multi-nozzle research track spray chamber (DeVries, Hollandale, MN)^44^ was then calibrated to simulate aerial applications of commercial insecticides recommended for adult WCR control. Two TT110015 nozzles spaced 0.76 m apart and 0.56 m above the target were used and the travel speed calculated according to the formula^45,46^:

$$Output\,per\,nozzle\,(L/min)=\frac{Volume\,application\,rate\,(L/ha)\times speed\,(km/h)\times nozzle\,spacing\,(m)\,}{600\,(correction\,factor\,for\,unit\,conversion)}$$

#### Efficacy of commercial insecticides

The aerial application simulation previously described was used to evaluate the performance of two commercial insecticides recommended for adult WCR control. Replicated experiments were repeated twice within a two-year period (2016/2017) in the research track spray chamber available in the PAT-Lab, North Platte, NE. The pyrethroid Brigade 2EC (bifenthrin 25.1%, FMC Corporation, Philadelphia, PA) was tested at the lowest and highest label rates recommended for rootworm control, 36.8 and 112.1 g a.i./ha respectively. The organophosphate Dimethoate 4EC (dimethoate 43.5%, Drexel Chemical Company, Memphis, TN) was tested at the lowest label rate of 369.9 g a.i./ha, which has been the common recommendation to provide WCR adult control in the US western Corn Belt. Insecticide solutions were prepared in tap water at the lowest and highest carrier volumes recommended for aerial application rates (18.7 and 46.8 L/ha, respectively).

The aerial spray coverage obtained in the cornfield at the Middle position was reproduced on 100 mm-diameter ×15 mm-height Petri dishes (Thermo Fisher Scientific Inc., Waltham, MA, Cat. No. FB0875713) that were pre-labeled and evenly distributed in the spray chamber to receive the insecticide applied to internal surfaces of dish bottoms and lids. Treatments were combinations of insecticide rate, carrier volume rate and WCR population. A total of four Petri dishes were used as replicates for each treatment, which were sprayed separately during four different rounds. Treated dishes were left opened for 30 min to ensure complete drying, then closed and transferred to cardboard boxes where they were kept in darkness at 23 ± 1 °C for a maximum of 16 h until use for WCR bioassays.

The WCR bioassays were conducted at the University of Nebraska-Lincoln, Lincoln, NE. In 2016, field-collected beetles were provisioned with ears of sweet corn for one week prior to testing whereas in 2017, the F_1_ generations of 2016 field-collected beetles already under lab rearing procedure were used. In both years, each Petri dish was infested with a group of 20 mixed-age beetles of even sex ratio. Active beetles were collected from rearing cages with a mouth aspirator and each group placed individually in 15 ml centrifuge tubes (VWR^®^, Radnor, PA - Cat. No. 76176-950). Beetles were anesthetized (1.5 minute inside a −20 °C freezer) and transferred to treated dishes. Four untreated dishes were used as controls for each WCR population tested and infested dishes were maintained under laboratory conditions of 23 ± 1 °C and 13 ± 1 h photophase. Mortality of beetles at each treatment combination was recorded after 24 h. Insects that did not respond to prodding or were unable to walk consistently when placed ventral side down were considered dead.

### Susceptibility of adult WCR populations to insecticide active ingredients

#### Chemicals

Analytical standards of bifenthrin, dimethoate and indoxacarb were used. Bifenthrin 98% was obtained from Chem Service Inc., West Chester, PA (Cat. No. N-11203-100MG/CAS: 82657-04-3). Dimethoate 99.5% and indoxacarb ≥95.0% were purchased from Sigma-Aldrich Corp., St. Louis, MO (Cat. No. 45449-100MG/CAS: 60-51-5 and 33969-25MG-R/CAS: 144171-61-9, respectively). The insecticides were dissolved and diluted in acetone ≥99.9% supplied by Sigma-Aldrich Corp (Cat. No. 650501/CAS: 67-64-1).

#### Bioassays

The susceptibility of adult WCR to insecticide active ingredients was estimated in 2017 when the F_1_ generation of beetles that were field-collected in 2016 started to emerge. The vial bioassay method previously adapted for WCR^5,13^ was used. The evaluation time and procedure were modified for this study as described below. Wheaton™ Glass 20 ml scintillation vials (Thermo Fisher Scientific Inc., Waltham, MA, Cat. No. 03-340-25N) were treated with 500 μl each of increasing concentrations of bifenthrin, dimethoate and indoxacarb diluted in acetone. For control, vials were treated with acetone only. The number of insecticide concentrations used to test each population varied from 5 to 7 depending on the number of insects available at the time. Insecticide concentrations were replicated three times for each WCR population tested. Vials were homogeneously coated internally and allowed to dry under a fume hood by rolling for 30 minutes at room temperature on a commercial roller machine (Nemco 8045SXW Hot Dog Roller Grill, Nemco Food Equipment Inc., Hicksville, OH). Each treated vial was infested with a group of ten 48h-old adults of even sex ratio. Vial caps were loosely closed to allow air exchange inside the vials while preventing beetle escape. After 24 h, one kernel of sweet corn was added to each vial to allow beetle feeding and at 48 h after infestation the mortality of beetles was recorded. Insects that did not respond to prodding or were unable to walk consistently when placed ventral side down were considered dead. All treatments were maintained at 23 ± 1 °C and 13 ± 1 h photophase.

### Statistical analysis

Corn canopy spray deposition rate and droplet size parameters reported from WSPs were subjected to analysis of variance in SAS 9.4 software (SAS Institute, Cary, NC) at significance level α = 0.05. The treatment design was a factorial arrangement with spray application rates (18.7 and 46.8 L/ha) and corn canopy position (Bottom, Middle, and Top) as factors in a randomized complete block experimental design (RCBD) where application lines were considered blocks.

To analyze the performance of commercial insecticides on adult rootworm populations under simulated aerial application conditions, insect mortality data collected from each treatment combination was corrected by Abbott’s formula for mean mortality in the respective untreated controls^47^. The corrected proportion mortality that has a continuous distribution within the restricted interval of [0,1] was analyzed with a Beta-binomial distribution using a logit link function with a generalized mixed model in SAS 9.4 software^48,49^. A completely randomized experimental design and factorial treatment design were used. Multiple comparison of treatment means was performed using Fisher’s least significant difference procedure at significance level α = 0.05.

The susceptibility of adult WCR populations to insecticide active ingredients was evaluated by analyzing the relationship between insecticide concentrations tested and mortality responses obtained. Data were corrected by Abbot’s formula for natural control mortality and analyzed with a probit link function with Normal distribution in POLOPlus-PC software (LeOra Software LLC)^50–52^. The probit procedure also estimated a Pearson goodness-of-fit chi-square value (χ^2^) testing the null hypothesis that the expected regression model adequately fits the data. Resistance ratios (RR_50_) with correspondent 95% confidence intervals (95%CI) were calculated by dividing the estimated LC_50_s of resistant populations by the estimated LC_50_ of each susceptible population of reference^53^.

## Results

### Aerial application simulation

#### Cornfield spray deposition

**Figure 1:**
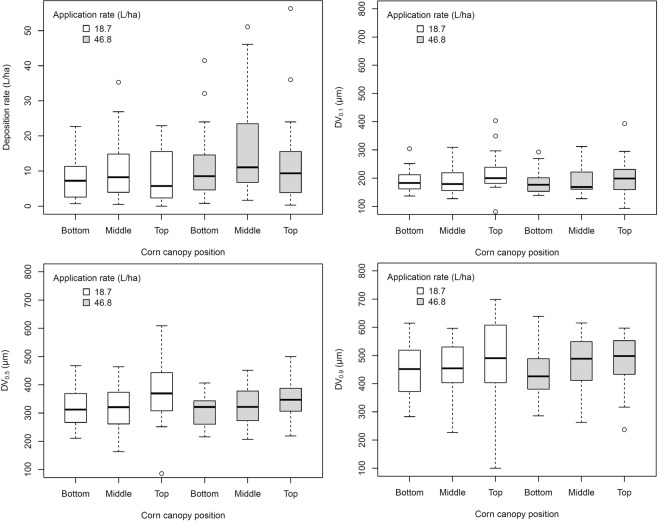
Field spray deposition rate and droplet size parameters (DV_0.1_, DV_0.5_, and DV_0.9_) collected from WSPs placed in the corn canopy at three positions denoted as Top, Middle, and Bottom prior to aerial application.

The spray deposition rates and droplet size characteristics obtained by aerial applications across corn canopies were considerably variable (Fig. 1). The WSP results indicated that the interaction between corn canopy position and application rate did not influence spray deposition rate (*F*_2,113_ = 0.11; *p* = 0.8999). Therefore, the interaction term was removed from the model. Application rate influenced spray deposition rate (*F*_1,115_ = 5.72; *p* = 0.0184), whereas corn canopy position did not (*F*_2,115_ = 1.42; *p* = 0.2450). The average deposition rate across the three corn canopy positions (Bottom, Middle, and Top) was within a 2.9–15.7 L/ha 95%CI for the aerial application performed at 18.7 L/ha, whereas the one performed at 46.8 L/ha resulted in average deposition values within a 7.3–20.1 L/ha 95%CI. The average deposition rates at Middle canopy position (used later for the spray-chamber calibration) were 10.9 and 16.5 L/ha for the lowest and highest aerial application rates evaluated, respectively.

The droplet size distribution reported in the WSP analysis indicated that the DV_0.5_ was not influenced by the interaction between corn canopy and application rate (*F*_2,113_ = 0.37; *p* = 0.6947), therefore the interaction term was removed from the model. Corn canopy alone influenced the DV_0.5_ of the spray deposition (*F*_2,115_ = 3.62; *p* = 0.0298), whereas application rate did not (*F*_1,115_ = 0.55; *p* = 0.4602). Estimated 95%CI for droplets deposited on the Top canopy position had greater DV_0.5_ (334–382 μm) when compared to Middle (299–346 μm) and Bottom (291–339 μm) canopy positions. The interaction between corn canopy position and application rate did not influence the DV_0.1_ (*F*_2,113_ = 0.32; *p* = 0.7279) or the DV_0.9_ (*F*_2,113_ = 0.31; *p* = 0.7356) of the spray deposition. Furthermore, both DV_0.1_ and DV_0.9_ were not affected by either application rate (*F*_1,115_ = 0.16; *p* = 0.6864 and *F*_1,115_ = 0.01; *p* = 0.9400, respectively) or corn canopy position (*F*_2,115_ = 2.73; *p* = 0.0696 and *F*_2,115_ = 1.34; *p* = 0.2659, respectively).

#### Wind tunnel and spray-chamber calibration

The wind tunnel test conducted in 2016 identified combinations of TT110015 nozzle and operating pressures compatible with the spray deposition data collected at Middle canopy position of corn (Fig. 1). The resultant spray-chamber calibration parameters and droplet size distribution for each chosen spray pressure combination are available in Table 1.

**Table 1:** Research track spray chamber application parameters used for aerial application simulations of commercial insecticides.

Application rate (L/ha)	Spray pressure (kPa)	Output per nozzle (L/min)	Application speed (Km/h)	Droplet size distribution (μm)		
				DV_0.1_	DV_0.5_	DV_0.9_
10.89	110.32	0.38 ± 0.003	27.25	188 ± 0.249	348 ± 0.358	528 ± 1.843
16.50	131.00	0.41 ± 0.003	19.68	178 ± 0.391	333 ± 0.745	506 ± 1.163

Output per nozzle and droplet size distribution data are mean values ± SE obtained from combinations of TT110015 nozzle and spray pressures that best replicated the cornfield aerial application deposition on the Middle canopy of corn.

#### Efficacy of commercial insecticides

**Figure 2:**
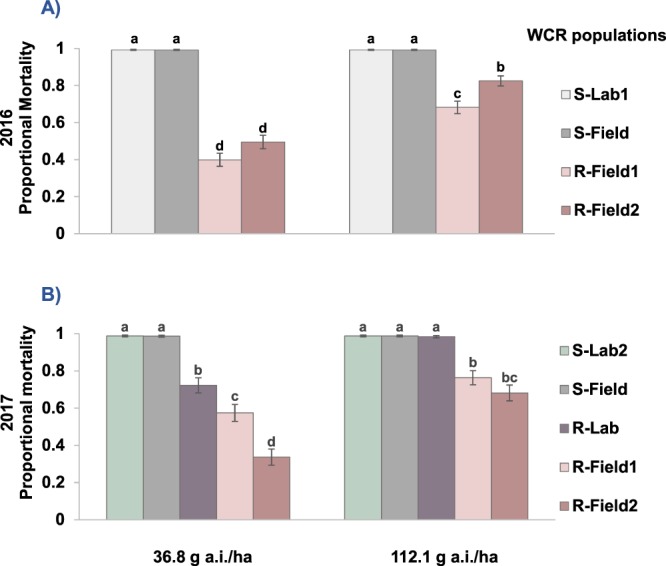
Efficacy of Brigade 2EC (25.1% bifenthrin) against pyrethroid-resistant (R-) and pyrethroid-susceptible (S-) western corn rootworm populations in simulated aerial application bioassays; (**A**) 2016 (*F*_3,56_ = 4.66, *p* = 0.0056), (**B**) 2017 (*F*_4,70_ = 8.48, *p* < 0.0001). Data are inverted link proportional mortality means ± SE. Within year and across two bifenthrin rates, treatment combination means followed by the same lower-case letter were not statistically different (Fisher’s LSD Test, *p* > 0.05).

Overall, there were no significant two- and three-way interaction effects of carrier volume on the performance of bifenthrin and dimethoate rates tested (*p* > 0.05), so this factor was excluded from the statistical model used. In both years, bifenthrin efficacy was significantly affected by the interaction of WCR populations and insecticide rates (Fig. 2). Mortality of resistant WCR populations was lower than susceptible populations over two rates of bifenthrin tested with a mean mortality range of 40–82% in 2016 and 34–76% in 2017. The highest label rate of bifenthrin was more effective against pyrethroid-resistant WCR than the lowest label rate tested. However, mortality of laboratory-selected resistant WCR (R-Lab) tested in 2017 was higher than mortality observed for field collected resistant WCR at both label rates of bifenthrin. In dimethoate bioassays, the lowest label rate (369.9 g a.i./ha) provided >99% mortality across all populations tested in the first run without statistical difference among treatments (*F*_4,35_ = 0.22, *p* = 0.9283) and 100% mortality in all treatments during the second run.

### Susceptibility of adult WCR populations to insecticide active ingredients

Probit regressions were obtained for WCR populations exposed to bifenthrin, dimethoate and indoxacarb active ingredients (Table 2). Values of χ^2^ obtained suggest that observed mortality data of tested populations fitted the expected probit regression model. Field populations tested that were considered resistant to pyrethroids (R-Field1, R-Field2 and R-Lab) exhibited reduced susceptibility to both bifenthrin and dimethoate active ingredients. Confidence intervals of resistance ratios (RRs) estimated for pyrethroid-resistant WCR populations overlapped for each insecticide tested and were different than those estimated for pyrethroid-susceptible populations (Table 2). However, RRs of pyrethroid-resistant WCR varied depending on the population used as the susceptible reference in the calculation. In general, higher RRs were obtained when using a non-diapausing lab susceptible population (S-Lab1 and S-Lab2) versus the field-collected susceptible population (S-Field). Compared to S-Lab2, RRs of pyrethroid-resistant populations ranged from 22.86- to 33.18-fold for bifenthrin and from 14.36- to 22.60-fold for dimethoate. Furthermore, compared to S-Lab2 and S-Field, pyrethroid-resistant WCR populations showed increased susceptibility to indoxacarb with RRs ranging from 0.32- to 0.35-fold (Table 2).

**Table 2:** Western corn rootworm adult susceptibility of pyrethroid-resistant (R-) and pyrethroid-susceptible (S-) populations estimated in 2017 for insecticide active ingredients.

Insecticide	Population	N^a^	Slope ± SE	LC_50_ (95% CI)^b^	χ² ^(d.f.)^	RR_50_ (95% CI)^c^		
Bifenthrin	S-Lab1	120	3.30 ± 0.53	0.19 (0.14–0.24)	1.19 (2)	1	—	—
	S-Lab2	180	1.57 ± 0.23	0.32 (0.19–0.47)	2.86 (4)	1.66 (1.00–2.76)	1	—
	S-Field	151	2.21 ± 0.36	2.49 (1.85–3.31)	0.42 (3)	13.08 (9.05–18.91)	7.89 (4.64–13.41)	1
	R-Lab	151	4.79 ± 1.21	7.23 (5.00–8.86)	1.65 (3)	37.92 (26.98–53.29)	22.86 (13.70–38.12)	2.90 (2.00–4.20)
	R-Field1	121	2.97 ± 0.53	7.55 (5.46–9.63)	0.33 (2)	39.60 (27.66–56.69)	23.87 (14.13–40.31)	3.03 (2.05–4.46)
	R-Field2	121	1.74 ± 0.40	10.49 (6.14–17.18)	0.79 (2)	55.05 (32.53–93.14)	33.18 (17.32–63.55)	4.21 (2.44–7.27)
Dimethoate	S-Lab2	150	3.29 ± 0.58	0.20 (0.15–0.24)	2.72 (3)	1	—	—
	S-Lab1	150	6.58 ± 1.82	0.37 (0.30–0.42)	1.57 (3)	1.87 (1.46–2.41)	1	—
	S-Field	120	11.75 ± 1.94	0.98 (0.92–1.05)	1.61 (2)	4.95 (3.96–6.18)	2.64 (2.28–3.06)	1
	R-Field1	181	3.09 ± 0.37	2.85 (2.33–3.47)	2.97 (4)	14.36 (10.74–19.21)	7.67 (6.05–9.72)	2.90 (2.36–3.57)
	R-Field2	153	3.41 ± 0.62	3.23 (2.36–4.06)	2.69 (3)	16.30 (11.68–22.76)	8.70 (6.52–11.61)	3.30 (2.53–4.29)
	R-Lab	121	2.57 ± 0.43	4.48 (3.44–5.64)	1.57 (2)	22.60 (16.40–31.15)	12.07 (9.18–15.86)	4.57 (3.57–5.85)
Indoxacarb	S-Lab2	152	2.66 ± 0.41	7.40 (3.90–11.19)	3.78 (3)		1	—
	S-Field	150	3.14 ± 0.58	7.62 (5.79–9.93)	0.28 (3)		1.03 (0.72–1.47)	1
	R-Field1	150	1.66 ± 0.31	2.47 (1.14–4.00)	2.81 (3)		0.33 (0.18–0.63)	0.32 (0.17–0.61)
	R-Lab	151	1.76 ± 0.27	2.58 (1.80–3.53)	1.66 (3)		0.35 (0.23–0.52)	0.34 (0.22–0.51)

^a^Number of insects tested; ^b^μg a.i./vial; ^c^resistance ratios relative to each S- population.

## Discussion

The method of aerial application simulation used in this study accurately reproduced aerial application parameters such as the combination of spray carrier volumes and droplet size distribution compatible to what is deposited on corn leaves in a field situation. This allowed a more realistic representation of aerial application than could be obtained with more traditional spray techniques often used in laboratory studies^54–56^. Although the wind tunnel calibration was performed with only one rate of formulated bifenthrin, droplet size deposition obtained could practically be used for other rates and insecticides. Previous research has shown that nozzle type has more influence on spray droplet size distribution than active ingredient and carrier solution^24^. Therefore, the droplet size distribution data collected worked as a useful baseline for the insecticide treatments tested in this study.

When used to evaluate the efficacy of formulated bifenthrin against WCR populations, the aerial application simulation consistently captured differences in performance among the rates tested. The significant difference in mortality between pyrethroid-resistant and control populations at both the lowest and highest label rates of formulated bifenthrin confirmed resistance to bifenthrin revealed in active ingredient bioassays and was consistent with anecdotal reports of reduced WCR adult control received from farmers and local crop consultants^13,14^. Conversely, the lowest label rate of formulated dimethoate provided optimal control of both pyrethroid-resistant and -susceptible WCR populations under simulated aerial application conditions despite the low level of dimethoate resistance that had been revealed when pyrethroid-resistant populations were bioassayed with active ingredients. Results suggest that the observed shift in dimethoate susceptibility of pyrethroid-resistant WCR populations did not reach a level that would lead to “practical resistance” defined as field-evolved resistance that reduces field efficacy of a pesticide with practical consequences for pest control^57^. This same phenomenon has been reported in other insect pest systems suggesting that the relationship between levels of resistance confirmed in dose-response bioassays and actual efficacy of formulated product in the field needs to be explored in order to understand the practical impact of resistance^58–61^.

The simulated aerial application method used was a conservative approach since it tested one model of spray deposition compatible with mid-canopy of corn plants of a given stage and beetles could not escape from treated surfaces. In the field there is a considerable interaction of environmental conditions, beetle movement behavior, and different levels of insecticide coverage within the corn canopy that could lead to differential adult exposure to insecticides. In fact, the spray deposition we collected in the field was fairly variable confirming the uneven canopy coverage previously observed for aerial applications^19^. Variable levels of WCR exposure to insecticide applications could potentially lead to greater survival of WCR beetles in the field than measured in the aerial application simulation method, which may contribute to evolution of insecticide resistance and increase resistance levels measured in the lab over time^62,63^.

Effective insecticide resistance management strategies include the integration of different control strategies and rotation of insecticide modes of action to reduce selection pressure and potential evolution of resistance^64,65^. However, compliance with management strategies depends on several social-economic aspects such as compatibility with growers’ tradition and past experiences, technology complexity, visibility of results, as well as technology cost and associated profitability^66^. Price and convenience often drive growers’ choice of pest management practices, which may lead to continued use of a specific insecticide^67,68^. The increase in off-patent generic bifenthrin formulations has lowered pricing of this insecticide often leading to grower preference over other products and frequent inclusion in tank mixtures with other pesticides. Also, regulatory action such as the Food Quality Protection Act has led to reevaluation and cancellation of many insecticides uses in agriculture^69^ so fewer modes of action remain available to manage WCR. Collectively, these factors probably have facilitated ongoing selection pressure and contributed to the evolution of WCR resistance to pyrethroids. Educational and incentive programs often fail to consider sociopolitical perspectives for effective resistance management and could be better improved by considering the farmers perception, the potential support of networked communities, and the compatibility between industry interests and federal policies^70^.

The recent EPA registration of indoxacarb for adult rootworm control in field corn^35^ provides a different mode of action that may be useful in WCR pyrethroid-resistance management programs. In this study, LC_50_’s from dose-response bioassays performed with indoxacarb active ingredient were lower for pyrethroid-resistant than -susceptible WCR populations. Also, calculated resistance ratios were <1.00, which suggests that pyrethroid-resistant WCR populations tested were more sensitive to indoxacarb than pyrethroid-susceptible populations. Indoxacarb is considered a pro-insecticide that needs bioactivation by esterase/amidase enzymes present in the target to become a more toxic compound^71^. Therefore, insecticide resistance mechanisms that involve increased activity of hydrolytic enzymes can result in a higher activation rate of indoxacarb in a negative cross-resistance relationship^71–^^73^ which could be further explored to manage pyrethroid-resistant WCR populations with enhanced metabolism. Our bioassay results suggest that the previously reported likely involvement of enhanced metabolism as part of the WCR pyrethroid-resistance mechanism^14^ may be contributing to increased susceptibility to indoxacarb.

In summary, we conclude that the simulated aerial application method used to evaluate the performance of formulated insecticides in corn effectively confirmed bifenthrin resistance and could be useful to evaluate the efficacy of various aerially-applied insecticides for other pest insects. Many of the insecticides still used in the US western Corn Belt belong to the pyrethroid class, so WCR resistance to bifenthrin and potential cross-resistance with other compounds significantly restricts control options in affected areas. Dimethoate and indoxacarb could be useful compounds to manage bifenthrin WCR resistance using rotation-based approaches. However, because of potential cross-resistance between structurally related pyrethroids and organophosphates^28–33^, dimethoate and other organophosphate insecticides should be used with caution in areas where WCR pyrethroid-resistance has been confirmed. In the western Corn Belt, WCR resistance evolution to rootworm-active *Bt* traits has increased the importance of aerially-applied insecticides and crop rotation as tactics to manage densities and mitigate resistance^74^. To develop more sustainable WCR management strategies, additional field research and modeling is needed to determine the biological and economical value of short- and long-term best management practices and specifically the optimal role of insecticides in the system. Because of the highly adaptable nature of WCR populations to selection pressure we reinforce the importance of using insect resistance management within an IPM framework to delay the evolution of WCR resistance and prolong the efficacy of formulated insecticide products and plant-incorporated traits.

## Data Availability

The datasets generated and /or analyzed during the current study are available from the corresponding author on reasonable request.
